# Polypharmacy Associated With the Simultaneous Prevalence of Dry Eye Due to Aqueous Deficiency and Hyposalivation in Adults Over 50 Years Old

**DOI:** 10.7759/cureus.76439

**Published:** 2024-12-26

**Authors:** Ximena Alejandra Checa-Caratachea, Álvaro Edgar González-Aragón Pineda, Myrna Miriam Valera-Mota, Aarón Bautista-Delgado, Gloria Alejandra Moreno-Altamirano, Luis Pablo Cruz-Hervert

**Affiliations:** 1 Master and Doctoral Program in Medical, Dental and Health Sciences - Faculty of Medicine, National Autonomous University of Mexico, Mexico City, MEX; 2 Faculty of Higher Studies Iztacala, National Autonomous University of Mexico, Mexico City, MEX; 3 Faculty of Medicine, National Autonomous University of Mexico, Mexico City, MEX; 4 Graduate Studies and Research Division at the Faculty of Dentistry, National Autonomous University of Mexico, Mexico City, MEX; 5 Department of Epidemiology, National Institute of Cardiology Ignacio Chávez, Mexico City, MEX

**Keywords:** aging, chronic diseases, comorbidity, dry eye, epidemiology, hyposalivation, multimorbidity, polypharmacy, xerostomia

## Abstract

Introduction

Dry eye and hyposalivation, often linked to Sjögren’s syndrome (SS), are prevalent among adults. However, systemic diseases and their associated medications also play a role, as drug interactions can intensify the effects of certain medications.

Objective

To assess whether polypharmacy is associated with the co-occurrence of aqueous-deficient dry eye (ADDE) and hyposalivation in adults aged 50 years and older without SS.

Methods

In a convenience sample of 455 adults who attended an optometry clinic, a medical history questionnaire was completed, and tear (Schirmer I) and salivary production (cotton weight test) were evaluated. To investigate the links between dry eye (due to aqueous deficiency and hyposalivation) and various factors (polypharmacy, chronic diseases, age, sex, education, marital status, employment, illicit drug, alcohol, and tobacco use), logistic regression modeling was employed. Then, odds ratios and 95% confidence intervals were obtained.

Results

A simultaneous prevalence of ADDE and hyposalivation of 16.7% (n = 76) (95% confidence interval (95% CI) 13.5%-20.4%) and a prevalence of polypharmacy of 23.1% (n = 105) (95% CI 19.4%-27.1%) were found. Subjects with polypharmacy had an 88.0% higher probability of developing ADDE and hyposalivation (OR = 1.88, 95% CI 1.07-3.29; p = 0.026).

Conclusions

Polypharmacy increases the likelihood of both ADDE and hyposalivation in individuals without SS. It is important to provide comprehensive and multidisciplinary care to adults, to detect these diseases in time, and to maintain strict control of the medications they need.

## Introduction

The tear and salivary glands are acinar exocrine glands responsible for producing enzymes, electrolytes, and proteins [1]. Both are innervated by the VII cranial nerve (facial nerve) through the parasympathetic system [2]. When there is a decrease in tear production, it is called aqueous-deficient dry eye (ADDE) if there is a decrease in tear production <10 mm/five minutes in Schirmer I [3]. The term hyposalivation is used to describe a decrease in salivary flow production, with levels dropping below 0.1 mL/minute at rest or less than 0.5 mL/minute when stimulated [4].

Approximately 5-50% of the global population has ADDE [5], while about 33.4% experience hyposalivation [6]. Simultaneous occurrence of dry eye from lack of tears and dry mouth has been observed in 4%-10% of cases [7-9]. Dry eye causes symptoms such as burning, blurred vision, hyperemia, tearing, gritty sensation, foreign body sensation, and itching [10]. At the same time, hyposalivation can cause symptoms such as halitosis, gingivitis, burning mouth sensation, caries, alteration in the taste system, and increased vulnerability to developing oral candidiasis, among others [11].

Autoimmune responses that target glandular tissue have been connected to both conditions, indicating a potential link to Sjögren’s syndrome (SS) [12]. However, ADDE and hyposalivation can occur for various reasons, among which are the presence of systemic diseases [8] and drug use [13]. When taking multiple drugs, as in polypharmacy, drug interactions can intensify the effects of certain medications, leading to nervous system signaling disruption through competition with neurotransmitters and alterations in tear and saliva production [4].

As we age, our bodies become more vulnerable to changes, and environmental factors like nutrition, physical activity, smoking, alcohol consumption, and sleep patterns contribute to the development of diseases requiring medication and potentially leading to polypharmacy [14].

According to the World Health Organization (WHO), polypharmacy is known as the intake of many drugs synchronously [15]. Some definitions of polypharmacy focus on the time drugs are taken, stating that it involves consuming two or more drugs for at least 240 days a year or five to nine drugs for at least 90 days [16]. In Mexico, a prevalence of polypharmacy of 15.5% has been estimated, and it was observed that this increases from the age of 50 [17]. Therefore, the objective of this study is to assess whether polypharmacy is associated with the co-occurrence of ADDE and hyposalivation in adults aged 50 years and older without SS. Our hypothesis is that polypharmacy may be associated with both, as it causes changes at the level of the nervous system that generate an alteration in the function of the exocrine glands, leading to a decrease in the production of tears and saliva [18].

## Materials and methods

A cross-sectional study took place from August 22, 2023, to May 27, 2024, involving adults 50 years of age and older who attended the Optometry Clinic at the UNAM’s Iztacala School of Higher Studies in Tlalnepantla de Baz, State of Mexico. The approval of the Ethics Committee of the Iztacala School of Higher Studies of the UNAM was obtained on March 8, 2022, with code CE/FESI/042022/1512. Adults who agreed to participate signed an informed consent form. This research complied with the ethical standards outlined by the Declaration of Helsinki and followed the Strengthening the Reporting of Observational Studies in Epidemiology (STROBE) Initiative guidelines for reporting epidemiological studies.

Participants

The study participants were drawn from the patient pool of the Optometry Clinic, which caters to roughly 2,314 individuals within the community, providing a range of eye care services such as refraction, contact lenses, visual rehabilitation, visual therapy, and diagnosis and treatment of eye diseases [19].

The Optometry Clinic belongs to the Iztacala Higher Education Faculty in the municipality of Tlalnepantla de Baz in the State of Mexico. Tlalnepantla de Baz is a municipality in the State of Mexico, with a total population of 672,202 inhabitants, of which about 28.8% are adults aged 50 years and older, and it also has an aging index of 87.3%. The literacy rate in Tlalnepantla de Baz is 98.0%, and the residents are mainly employed in the private sector, manufacturing, commerce, transportation services, and construction [20].

A sample size calculation was performed for logistic regression, with a prevalence of dry eye and hyposalivation of 2% in adults not exposed to polypharmacy [7], a proportion of non-exposed to polypharmacy of 67%, to detect an odds ratio of 5.7 [8], a statistical power of 80%, with a non-response rate of 20%, resulting in n = 348 subjects. To bolster the robustness of our findings, we included data from 455 individuals, leading to a 97.2% power in our sample size. The sampling was done using a non-probabilistic quota method. Quota sampling was carried out by selecting all participants who attended the Optometry Clinic and met the age criterion of 50 years or older at the time of the study.

For the inclusion criteria, all adults of both sexes aged 50 years and older were considered. As exclusion criteria, all subjects with a previous diagnosis of SS and with suspicion of SS, according to the screening obtained from the Profile of Fatigue and Discomfort - Sicca Symptoms Inventory (PROFAD-SSI)-short form index [21], were considered (Table 1). First, questions were asked to find out if the participant had a previous diagnosis of SS (yes/no), rheumatoid arthritis (yes/no), or systemic lupus erythematosus (yes/no). Participants answering “yes” to any of the three questions were excluded. If he or she answered negatively, the questions based on the index were applied. Exclusion criteria included answering affirmatively to all five questions for women and four for men.

**Table 1:** Screening questions applied to participants to rule out Sjögren's syndrome

Question	Answer
If the individual answers “Yes” to any of the following questions, the individual is excluded.	
Have you been diagnosed with rheumatoid arthritis?	Yes/no
Have you been diagnosed with systemic lupus erythematosus?	Yes/no
Have you been diagnosed with Sjögren's syndrome?	Yes/no
If the individual answers “Yes” to all five questions, the individual is excluded.	
Have you experienced discomfort in your limbs? For example: discomfort in your joints (such as hips, knees, or shoulders), in your muscles, or general pain.	Yes/no
Have you had swelling in your fingers or wrists?	Yes/no
Have your hands ever felt uncomfortably cold?	Yes/no
Have you been experiencing dry, itchy skin?	Yes/no
In case the individual is a woman	
Have you had vaginal dryness?	Yes/no

Those with a previous diagnosis of rheumatoid arthritis or systemic lupus erythematosus, wearers of ocular prostheses in at least one eye, who at the time of the study had conjunctivitis, with recent eye surgeries, with eyelid alterations, who wore contact lenses at the time of the study, and who had a history of radiation to the head and/or neck were also excluded.

Variables

The dependent variable was considered as the diagnosis of ADDE (tear production <10 mm for five minutes in both eyes [3]) and hyposalivation (production ≤0.1 mL/minute [4]) simultaneously. The independent variables were the presence of polypharmacy (concomitant consumption of three drugs or more for at least six months), chronic diseases (diabetes mellitus, arterial hypertension, heart disease, lung disease, anxiety and/or depression (yes/no)), age (years completed), and sex (female/male). Other variables considered were educational level (basic (six years of education)/middle and high school (12 years of education)/higher education (with university degree)), marital status (without partner (married and domestic partnership)/with partner (single, divorced and widowed)), employment status (unemployed or retired/active worker), illicit drug use (yes/no), alcohol (yes/no) and tobacco (yes/no).

Data collection

Data collection was carried out from 8 am to 2 pm to reduce the influence of circadian variations. Participants were asked not to have consumed food, drinks, chewing gum, candy, or tobacco at least one hour before the study. Data collection was carried out in an office within the Optometry Clinic.

Questionnaire Application

All participants received a 41-question survey about their background, health, illicit drug use, and risk factors. Content validity was assessed through expert judgment (five physicians and three dentists). Internal consistency and reliability were assessed, obtaining a Cronbach’s alpha of 0.87 and an intraclass correlation of 0.97, respectively. The first section of the questionnaire consisted of eight questions about patient identification data, including questions about name, age, sex, marital status, educational level, and occupation. The second section asked about the participants’ medical history, which included questions about the diagnosis of systemic diseases and illicit drug use, inquiring about the type, number of drugs, and time of use. The use of eight different types of medications was questioned: anti-inflammatory drugs, diuretics, hypotensive drugs, hypoglycemic drugs, antidepressants, anxiolytics, antihistamines, and antispasmodics. The questionnaire’s last section focused on electronic device usage and alcohol, tobacco, and drug consumption, asking participants how often they used or consumed each.

To obtain data on medication use, each participant was asked the question, “Have you taken medication regularly in the last six months? For example: multivitamins, food supplements, paracetamol, ibuprofen, etc.” If the patient answered affirmatively, the medications mentioned were noted down, as well as the time taken for each one, and then the questions “How many medications do you take a day?” and “How many pills do you take a day?” were asked. It was then verified that the number of medications that the participant indicated they took a day matched the types of medications reported; if not, the medications taken were investigated. Finally, polypharmacy was categorized according to the number of medications that each participant reported taking.

Evaluation for Diagnosis of ADDE and Hyposalivation

Using Schirmer I (for ADDE) and cotton weight sialometry (for hyposalivation) as gold standards, four standardized examiners conducted the evaluation. During the evaluation, the participant was seated, with his head upright and looking straight ahead. The evaluation consisted of applying the Schirmer I test for ADDE, for which calibrated filter paper strips were placed on the outer corner of each eye [22]. The measurement on the Schirmer strip was done visually, noting the millimeters of tears absorbed based on the amount of strip that was moistened. The cotton weight test consisted of placing a cotton swab on the floor of the mouth below the tongue of each individual [23], using a calibrated scale (Series YS™; OHAUS Corporation, Parsippany, NJ). The cotton swab was weighed before and after being placed on each patient; 1 g of weight was considered equivalent to 1 mL of saliva. Both the Schirmer strips and the cotton were placed at the same time. When they were placed, the stopwatch was activated, and after five minutes, both strips and the cotton were removed to record the data. To consider a positive diagnosis of ADDE and hyposalivation, the measurement of tear production <10 mm for five minutes in both eyes [3] and resting saliva production ≤0.1 mL/minute, respectively [4], was considered.

Data analysis

Data capture was carried out using the EpiData 3.1 software (EpiData Association, Odense, Denmark), and statistical analysis was carried out using Stata 17 software (StataCorp LLC, College Station, TX). A descriptive analysis of the data was performed, obtaining the frequencies and percentages of the variables evaluated. The prevalence of ADDE and hyposalivation was calculated simultaneously with their 95% confidence intervals. A bivariate analysis was then carried out using chi-square and Fisher's exact test between the dependent variable (ADDE) and hyposalivation simultaneously) and the independent variables (polypharmacy, chronic diseases, age, sex, educational level, marital status, employment status, illicit drug, alcohol, and tobacco use). A p-value <0.05 was considered statistically significant.

A multivariate analysis was carried out using binary logistic regression, where those variables with a p-value of <0.25 and those that were important to include due to biological plausibility were included. Logistic regression was used to obtain prevalence odds ratios and calculate 95% confidence intervals, taking as statistically significant those values where the unit was not included in the confidence interval and p-values <0.05. Finally, the presence of interactions was sought. 

## Results

**Figure 1:**
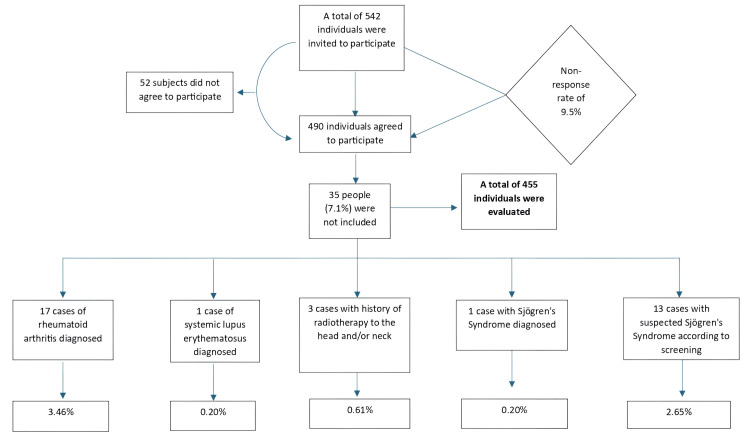
Non-response rate and exclusion criteria among the participants

A total of 542 individuals were invited to participate, of which 490 agreed to participate, resulting in a non-response rate of 9.5%. The main reasons why individuals did not agree to participate were lack of time and lack of interest. After applying exclusion criteria, 35 participants were removed from the study, resulting in a final sample size of 455 (Figure 1). The participants were aged 50-92 years, with a median age of 64 years (mean = 63.6, standard deviation = 8.9). Most participants were 60 or older (64.2%), and 56.0% were female.

Prevalence of ADDE and hyposalivation

**Figure 2:**
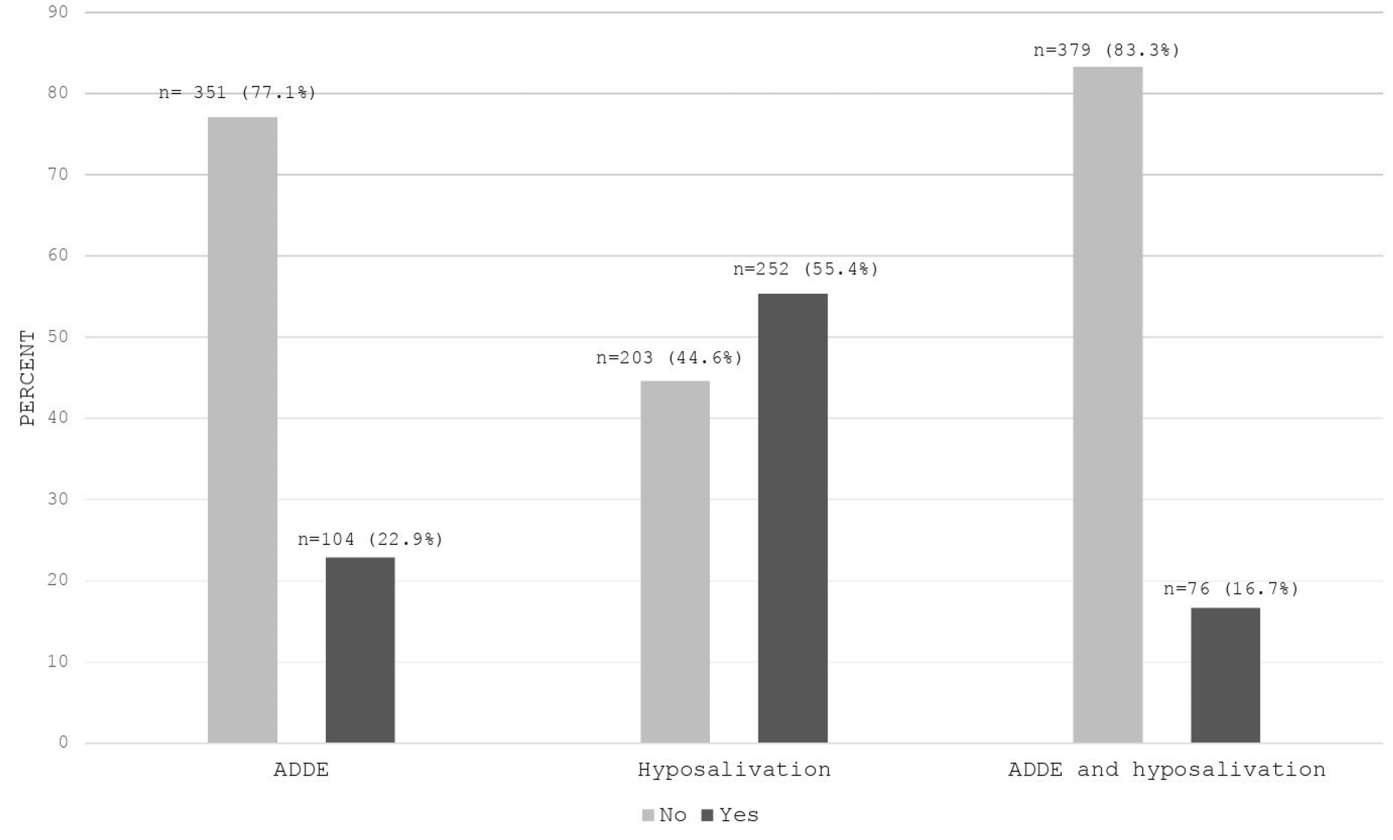
Prevalence of dry eye due to aqueous deficiency and hyposalivation simultaneously in adults over 50 years old (n = 455) ADDE = aqueous-deficient dry eye

A prevalence of ADDE of 22.9% (95% CI 19.2%-26.9%) was found (n = 104), the prevalence of hyposalivation was 55.4% (95% CI 50.7%-59.0%) (n = 252), and the prevalence of ADDE and hyposalivation simultaneously was 16.7% (95% CI 13.5%-20.4%) (n = 76) (Figure 2). 

Risk indicators

This study found no link between sociodemographic factors, chronic illnesses, smoking, alcohol or illicit drug use, and the simultaneous presence of ADDE and hyposalivation (p > 0.05) (Tables 2-3). 

**Table 2:** Prevalence of dry eye due to aqueous deficiency and hyposalivation simultaneously according to sociodemographic variables ADDE = aqueous-deficient dry eye; *with neither of the two conditions or only one: ADDE or hyposalivation; **chi-square test; ***basic = six years of education/middle and high school = 12 years of education/higher = with a university degree.

Variable	* No ADDE + hyposalivation n = 379 (%)	With ADDE + hyposalivation n = 76 (%)	Total n = 455 (%)	p**
Age				
50-59 years old	141 (86.5)	22 (13.5)	163 (100.0)	-
> 60 years old	238 (81.5)	54 (18.5)	292 (100.0)	0.171
Sex				
Men	168 (84.0)	32 (16.0)	200 (100.0)	-
Women	211 (82.8)	44 (17.2)	255 (100.0)	0.722
Educational level***				
Basic	228 (83.8)	44 (16.2)	272 (100.0)	-
Middle and high school	75 (79.0)	20 (21.0)	95 (100.0)	0.379
Higher education	76 (86.4)	12 (13.6)	88 (100.0)	-
Marital status				
Without a partner	127 (85.8)	21 (14.2)	148 (100.0)	-
With a partner	252 (82.1)	55 (17.9)	307 (100.0)	0.318
Employment status				
Unemployed or retired	241 (81.1)	56 (18.9)	297 (100.0)	-
Employed	138 (87.3)	20 (12.7)	158 (100.0)	0.092

**Table 3:** Prevalence of dry eye due to aqueous deficiency and hyposalivation simultaneously due to chronic diseases, tobacco, alcohol, and illicit drug use ADDE = aqueous-deficient dry eye; *with neither condition nor only one: ADDE or hyposalivation; **chi-square test; ***Fisher's exact test.

Illness or substance abuse	*No ADDE + hyposalivation n = 379 (%)	With ADDE + hyposalivation n = 76 (%)	Total n = 455 (%)	p**
Diabetes mellitus				
No	263 (85.1)	46 (14.9)	309 (100.0)	-
Yes	116 (79.5)	30 (20.5)	146 (100.0)	0.131
Heart disease				
No	352 (83.8)	68 (16.2)	420 (100.0)	-
Yes	27 (77.1)	8 (22.9)	35 (100.0)	0.310
Depression				
No	347 (83.2)	70 (16.8)	417 (100.0)	-
Yes	32 (84.2)	6 (15.8)	38 (100.0)	0.875
Anxiety				
No	344 (84.1)	65 (15.9)	409 (100.0)	-
Yes	35 (76.1)	11 (23.9)	46 (100.0)	0.167
Tobacco use				
No	325 (82.1)	71 (17.9)	396 (100.0)	-
Yes	54 (91.5)	5 (8.5)	59 (100.0)	0.069
Consumption of alcoholic beverages				
No	266 (81.6)	60 (18.4)	326 (100.0)	-
Yes	113 (87.6)	16 (12.4)	129 (100.0)	0.122
Illicit drug use***				
No	376 (83.2)	76 (16.8)	452 (100.0)	-
Yes	3 (100.0)	0 (0.0)	3 (100.0)	0.436

Regarding medication use, an association was found between the simultaneous presence of ADDE and hyposalivation and the use of antihypertensives. The prevalence found was 23.3% in those who use antihypertensives versus 12.8% in those who do not use them (p = 0.004).

A prevalence of polypharmacy of 23.1% (95% CI 19.4%-27.1%) was found (n = 105). Polypharmacy was significantly associated with a higher prevalence of ADDE and dry mouth, with 24.8% of polypharmacy patients experiencing both conditions compared to 14.3% of those without polypharmacy (p = 0.012) (Table 4).

**Table 4:** Prevalence of dry eye due to aqueous deficiency and hyposalivation simultaneously according to the medication consumed and polypharmacy ADDE = aqueous-deficient dry eye; *with neither condition nor only one: ADDE or hyposalivation; **Fisher's exact test; ***chi-square test.

Type of drug	*No ADDE + hyposalivation n = 379 (%)	With ADDE + hyposalivation n = 76 (%)	Total n = 455 (%)	p**
Diuretics				
No	364 (83.87)	70 (16.1)	434 (100.0)	-
Yes	15 (71.4)	6 (28.6)	21 (100.0)	0.139
Antidepressants				
No	367 (83.4)	73 (16.6)	440 (100.0)	-
Yes	12 (80.0)	3 (20.0)	15 (100.0)	1.000
Anxiolytics				
No	362 (84.2)	68 (15.8)	430 (100.0)	-
Yes	17 (68.0)	8 (32.0)	25 (100.0)	0.050
Hypotensive***				
No	251 (87.2)	37 (12.8)	288 (100.0)	-
Yes	128 (76.7)	39 (23.3)	167 (100.0)	0.004
Hypoglycemic agents***				
No	274 (84.8)	49 (15.2)	323 (100.0)	-
Yes	105 (79.6)	27 (20.4)	132 (100.0)	0.170
Antispasmodics				
No	376 (83.2)	76 (16.8)	452 (100.0)	-
Yes	3 (100.0)	0 (0.0)	3 (100.0)	1.000
Antihistamines				
No	364 (83.3)	73 (16.7)	437 (100.0)	-
Yes	15 (83.3)	3 (16.7)	18 (100.0)	1.000
Polypharmacy***				
No	300 (85.7)	50 (14.3)	350 (100.0)	-
Yes	79 (75.24)	26 (24.8)	105 (100.0)	0.012

Multivariate analysis

The logistic regression model identified sex, age, smoking, educational level, depression, and polypharmacy as key factors associated with ADDE and hyposalivation. The Pearson chi-square goodness of fit test showed that the model correctly describes the data with a chi-square value of 203.6 (p = 0.734). Adults with these factors had a significantly higher probability (88.0%) of developing this condition compared to those without polypharmacy (OR = 1.88; 95% CI 1.07-3.29; p = 0.026) (Table 5). No statistically significant interactions were found.

**Table 5:** Logistic regression model for dry eye due to aqueous deficiency and hyposalivation simultaneously OR = odds ratio; 95% CI = 95% confidence interval; reference: sex = male; age = years; smoking = no; educational level = basic; depression = no; polypharmacy = no.

Variable	Raw OR		Adjusted OR	
	OR (CI 95%)	p	OR (CI 95%)	p
Sex	1.09 (0.66-1.80)	0.722	1.06 (0.62-1.81)	0.819
Age	1.03 (1.00-1.06)	0.016	1.02 (1.00-1.05)	0.050
Tobacco use	0.42 (0.16-1.09)	0.077	0.43 (0.16-1.13)	0.090
Educational level	0.74 (0.38-1.45)	0.392	0.72 (0.36-1.45)	0.373
Depression	0.92 (0.37-2.30)	0.875	0.76 (0.29-1.96)	0.579
Polypharmacy	1.97 (1.15-3.37)	0.013	1.88 (1.07-3.29)	0.026

## Discussion

This study found that 16.7% (95% CI 13.5%-20.4%) of participants experienced both ADDE and hyposalivation. In addition, a prevalence of ADDE and hyposalivation simultaneously in adults with a polypharmacy of 24.8% (p = 0.012) was found. Adults with polypharmacy have an 88% higher probability of developing ADDE and hyposalivation compared to adults without polypharmacy (OR = 1.88; 95% CI 1.07-3.29; p = 0.026). Several studies evaluate dry eye and hyposalivation in patients with SS, but there are few studies outside of SS that evaluate dry eye and hyposalivation simultaneously, so the main strength of the present study is that it is the first one carried out in Mexico and the first to evaluate ADDE and hyposalivation simultaneously that seeks to establish an association with polypharmacy.

This study distinguishes itself by clinically measuring tear and saliva production, unlike other studies that rely solely on reported symptoms, such as the one by Villa et al. [24], which only assessed dry mouth and dry eye sensations. While Wang et al. [9] utilized the Xerostomia Inventory questionnaire, a validated instrument, to assess dry mouth, their study did not employ gold standard tests like the Schirmer I test for dry eye, which measures aqueous deficiency. Conversely, while Fostad et al. [25] and Schein et al. [8] employed Schirmer I to assess tear production, their dry mouth evaluations relied solely on subjective reports of dry mouth sensation.

A key advantage of this study is that both tears and saliva production were measured in the morning, unlike previous research like Hynne et al. [7], which assessed saliva in the morning and tears in the evening. This is important because the circadian rhythm causes tear production to be greater during the morning and midday but lower during the afternoon and evening [26]. The same occurs with saliva production, with greater amounts produced during the day than in the afternoon and evening [27]. The main limitation of this study is that it was carried out in a clinic, which affects the external validity due to the presence of a Berkson bias or selection bias, in which participants are exposed to certain risk factors and events that lead them to request a health service [28]. Logistic regression was used to mitigate potential bias in the statistical analysis. However, it is important to take this into consideration when extrapolating the results of this study to the general population, then, logistic regression was used to mitigate potential bias in the statistical analysis.

The prevalence found in the present study is higher than that reported by Hynne et al. at 4% [7], Schein et al. at 4.4% [8], and Wang et al. at 10.0% [9]. The discrepancy in prevalence could be attributed to the diverse evaluation methods used to diagnose dry eye and hyposalivation, as Hynne’s study incorporated objective and subjective assessments of tears and saliva and focused on a specific age group of adults over 65. This research evaluated non-stimulated salivary flow in adults over 50, unlike previous studies that considered both stimulated and non-stimulated flow. Schein’s study investigated dry mouth symptoms; however, hyposalivation was not objectively analyzed. In contrast, the absence of objective evaluation’s technical attributes in subjective instruments may lead to either overdiagnosis (false positives) or underdiagnosis (false negatives).

The present study found that the presence of polypharmacy increased the odds of presenting ADDE and hyposalivation in adults. The association between polypharmacy and decreased tear and saliva production, even without xerostomic drugs, might be explained by the exocrine nature of the salivary and tear glands. Therefore, their functioning is governed by the parasympathetic autonomic nervous system and the consumption of several medications, increasing the use of inappropriate medications that can cause adverse effects due to drug interactions, for example, the pharmacodynamic drug-drug interaction in which there is a synergy or a potentiation of the effect of the medications, which could alter the receptors. In addition, the physiological functions of the human being change as age advances, which increases the sensitivity of the therapeutic effects; on the other hand, it is important to keep in mind that drugs compete with neurotransmitters, causing a blockage of signaling that leads to a decrease in the production of secretions [18].

Polypharmacy is a multifactorial problem. Factors contributing to its appearance include adverse drug reactions due to improper prescriptions or poor adherence to treatment, leading patients to self-medicate with over-the-counter remedies for symptoms like headache, nausea, and vomiting. Conversely, this could prompt them to seek treatment from their doctor or specialists, resulting in more drug prescriptions for symptom management. This factor is known as the “prescription cascade” [29].

As a consequence of the above, self-medication and inappropriate prescription of medications are considered an important risk factor for the development of polypharmacy due to the vulnerability of older adults to develop chronic diseases, in addition to the fact that pharmaceutical advertising influences the population by leading them to self-medicate in the presence of any symptom [30]. It is essential to carry out future longitudinal studies to establish temporality, which would allow us to see other factors that intervene in the development of these conditions. It is also crucial to understand how ADDE and low saliva production together affect people’s quality of life.

Limitations of the study

Because participants were non-probabilistically sampled from an Optometry Clinic and thus differ systematically from the general population (e.g., health and medication use), the study’s findings may be limited by selection bias and lack generalizability. Another limitation of the study is that obtaining information on the history of chronic diseases, medication use, and lifestyle of the participants in a self-reported manner, that is, through a questionnaire, may introduce a recall bias since the information may be inaccurate. A further limitation is that despite the effort to control the variables that are potential confounders through statistical adjustment in the model, residual confusion may exist. Generalizing the study’s findings might be difficult, as participants were limited to adults aged 50+ from one geographic area, which may not represent other age groups or populations. On the other hand, the statistical analysis was performed by categories and the sample size in some categories may be insufficient to reliably detect effects. Finally, it is important to mention that despite having found an association between polypharmacy and ADDE and hyposalivation simultaneously, further research is needed on possible biological pathways that explain the underlying mechanisms for establishing such an association.

## Conclusions

Our findings reveal a notable concurrence of ADDE and hyposalivation in individuals without SS, and polypharmacy emerges as a key factor associated with this phenomenon. It is important to pay more attention to medication in adults and raise awareness about the indiscriminate use of drugs. Furthermore, treating adult symptoms is crucial for enhancing quality of life, as dry eye linked to aqueous deficiency has been found to be a risk factor for developing hyposalivation and vice versa, emphasizing the need for multidisciplinary care.
